# Serious gastric perforation after second stereotactic body radiotherapy for peripheral lung cancer that recurred after initial stereotactic body radiotherapy: a case report

**DOI:** 10.1186/s13256-017-1504-z

**Published:** 2017-12-10

**Authors:** Hotaka Nonaka, Hiroshi Onishi, Masatoki Ozaki, Kengo Kuriyama, Takafumi Komiyama, Ryo Saito

**Affiliations:** 1 Department of Radiology, University of Yamanashi, 1110 Shimokato, Chuo City, Yamanashi 409-3898 Japan; 2 Department of Radiation Oncology, Shizuoka City Shimizu Hospital, 1231 Miyakami, Shimizu Ward, Shizuoka City, Shizuoka 424-8636 Japan; 3 Department of Radiology, Shizuoka General Hospital, 4-27-1 Kitaando, Aoi Ward, Shizuoka City, Shizuoka 420-8527 Japan

**Keywords:** Stereotactic body radiotherapy, Re-irradiation, Lung cancer, Gastric perforation

## Abstract

**Background:**

In recent reports, re-irradiation with stereotactic body radiotherapy for lung tumors in patients previously treated with thoracic radiation therapy resulted in several serious toxicities. Serious non-lung toxicities were observed mostly in patients with central tumors, but we experienced a case of fatal gastric perforation after a second stereotactic body radiotherapy in a patient with a peripheral lung tumor.

**Case presentation:**

An 83-year-old Asian man was diagnosed with T2N0M0 lung cancer in the form of squamous cell carcinoma in the lower lobe of his left lung. He was treated with stereotactic body radiotherapy of 40 Gy in 4 fractions and the tumor decreased in size in partial response. The local tumor recurred 8 months after the first stereotactic body radiotherapy, and he was re-irradiated with a second stereotactic body radiotherapy of 50 Gy in 4 fractions. A Sengstaken–Blakemore tube was inserted below his diaphragm by laparoscopic surgery before the second stereotactic body radiotherapy in order to reduce the stomach dose by keeping his stomach apart from the tumor. Two months after the second stereotactic body radiotherapy, he developed fatal gastric perforation and gastropleural fistula penetrating his diaphragm.

**Conclusions:**

To the best of our knowledge, this is the first report about a gastric perforation after stereotactic body radiotherapy for lung tumors and it warns of serious complication of stereotactic body radiotherapy in not only centrally located but also peripherally located tumors like in this case.

## Background

Stereotactic body radiotherapy (SBRT) plays a major role in the treatment of early-stage non-small cell lung cancer and oligometastatic lung tumors [1]. In recent reports, SBRT has also been performed for lung tumors in patients previously treated with thoracic radiation therapy [2–17]. Although most toxicities after re-irradiation with SBRT for these patients were grade 3 or less, several reports showed serious toxicities of grade 4 to 5 [4, 6, 7, 10, 11, 17] (Table 1). In these reports, most serious non-lung toxicities were observed in cases with central lung tumors. However, we experienced a case of fatal gastric perforation after the second SBRT for a peripheral lung tumor that recurred after the first SBRT.

**Table 1:** Serious toxicities after re-irradiation with stereotactic body radiotherapy for lung tumors in patients previously treated with thoracic radiotherapy

Authors and Reference number	Number of patients	Tumor location (central/peripheral)	Initial irradiation dose^a^	Re-SBRT dose^a^	Non-lung toxicities of grade 4–5 (tumor location)	Lung toxicities of grade 4–5
Peulen *et al*. [4]	29	11/21	30 Gy/2 Fr 40 Gy/4 Fr	30 Gy/2 Fr 40 Gy/5 Fr	G5 hemoptysis: 3 pts (central) G4 others^b^: 2 pts (central)	None
Liu *et al*. [6]	72	4/68	63 Gy/Conv	50 Gy/4 Fr	NA	G5 pneumonitis: 1 pt
Reyngold *et al*. [7]	39	NA	61 Gy/Conv	70.4 Gy (BED_10_)	G4 skin: 1 pt^c^ (peripheral)	None
Kilburn *et al*. [10]	33	17/16	66 Gy/33 Fr 50 Gy/5 Fr	50 Gy/10 Fr	G5 aorta-esophageal fistula: 1 pt (central)	None
Trovo *et al*. [11]	17	17/0	50–60 Gy/20–30 Fr	30 Gy/5–6 Fr	G5 hemoptysis: 1 pt (central)	G5 pneumonitis: 1 pt
Parks *et al*. [17]	27	18/11	64.8 Gy/Conv	50 Gy/5 Fr	G4 chest wall pain: 1 pt	None

*Abbreviations*: *BED*_*10*_ biologically effective dose (α/β = 10), *Conv* conventional fractionation, *Fr* fractions, *G* grade, *NA* not available, *pt* patient, *Re-SBRT* re-irradiation with stereotactic body radiotherapy

^a^Median dose or frequently used dose

^b^Vena cava superior stenosis and fistula between the trachea and gastric tube developed in a case with a recurrent tumor at the carina

^c^SBRT was performed for a right lung tumor in a patient who had received contralateral lung irradiation [19]

## Case presentation

An 83-year-old Asian man with a left lung tumor was referred to our hospital for SBRT in 2008. He had no clinical symptoms. Chest computed tomography (CT) showed a 3.5 cm-sized tumor located close to his left diaphragm. Magnetic resonance imaging of his head and fluorodeoxyglucose-positron emission tomography (FDG-PET)/CT showed no metastatic lesions. The maximum standardized uptake value (SUVmax) of the tumor on FDG-PET/CT was 6.9. The serum cytokeratin-19 fragment (CYFRA 21-1) level was elevated to 8.7 ng/mL. A transbronchial lung biopsy revealed squamous cell carcinoma. The lung cancer was staged at T2N0M0 based on the sixth TNM classification of malignant tumors. He had undergone upper lobectomy for stage I squamous cell lung cancer in 1993, and his medical history also included hypertension, atrial fibrillation, renal sclerosis, and chronic bronchitis with low respiratory function. Radical left lower lobectomy was not applicable because of his advanced age, history of left upper lobectomy, and low respiratory function; therefore, he selected SBRT.

**Fig. 1:**
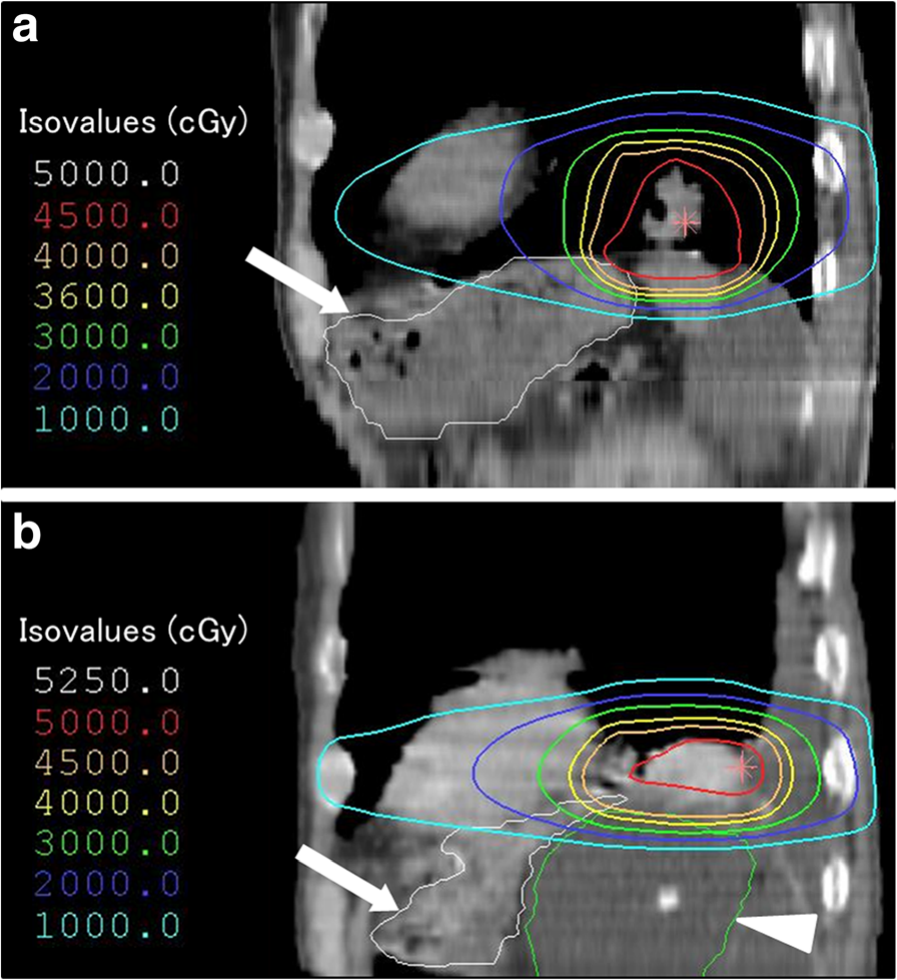
Dose distributions on sagittal images in stereotactic body radiotherapy. **a** The first stereotactic body radiotherapy. **b** The second stereotactic body radiotherapy. The white line (*arrow*) and green line (*arrowhead*) show the stomach and the balloon of the Sengstaken–Blakemore tube, respectively

**Fig. 2:**
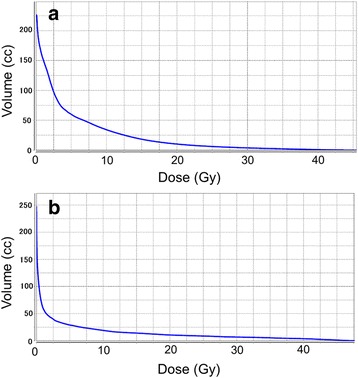
Dose volume histograms of the stomach in stereotactic body radiotherapy. **a** The first stereotactic body radiotherapy. **b** The second stereotactic body radiotherapy

SBRT was performed with the self-breath-holding technique using a respiratory monitoring system. Image guidance was performed using a CT-on-rails system for all sessions. A total dose of 40 Gy in 4 fractions was delivered to 95% of the planning target volume. The details of irradiation are shown in Table 2. The maximum dose (Dmax) in the stomach volume was 45.8 Gy. The dose distribution and dose volume histogram of his stomach are shown in Figs. 1a and 2a, respectively.

**Table 2:** Details of irradiations in the present case

	First SBRT	Second SBRT
Total dose (Gy)	40	50
Fractions	4	4
Prescription	PTV D95	Isocenter
Target volume	CTV = GTV	CTV = GTV
	ITV = CTV + 1 mm	ITV = CTV + 4 mm
	PTV = ITV + 5 mm	PTV = ITV + 5 mm
Leaf margin (mm)	5	0
Beam energy (MV)	6	6
Beam arrangement	Non-coplanar dynamic arcs	Non-coplanar dynamic arcs
Dose calculation	Superposition	Superposition

*Abbreviations*: *CTV* clinical target volume, *GTV* gross tumor volume, *ITV* internal target volume, *PTV D95* dose to 95% of the planning target volume, *SBRT* stereotactic body radiotherapy

**Fig. 3:**
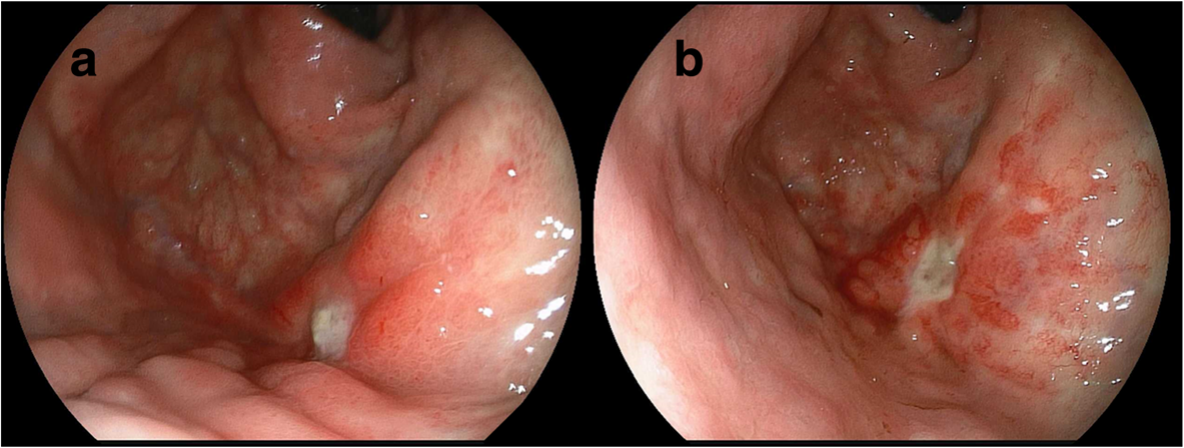
Endoscopic images of stomach after the first stereotactic body radiotherapy. A deep ulcer on the fornix was observed at 3 (**a**) and 8 months (**b**) after the first stereotactic body radiotherapy

Immediately after the completion of SBRT, he developed vomiting and upper abdominal pain, and he was consequently treated with a histamine H2-receptor antagonist. Two months after SBRT, CT revealed a partial response of the lung tumor, and the serum CYFRA 21-1 level had decreased to the normal range. However, approximately 3 months after SBRT, he developed hematemesis. Endoscopy showed a deep ulcer on the fornix of his stomach (Fig. 3a), and he was treated with a proton pump inhibitor. Eight months after SBRT, repeated endoscopy showed an intractable ulcer (Fig. 3b). Around the same time, local recurrence was diagnosed by tumor progression on CT images accompanying high uptake on FDG-PET/CT (SUVmax, 4.9) and elevation of the serum CYFRA 21-1 level. A 4.5 cm-sized recurrent tumor was observed, in contact with his left diaphragm, while metastatic disease was not noted. The recurrent tumor did not cause any symptoms. He did not accept the risk of salvage segmentectomy, and radiofrequency ablation was not applicable because of the large tumor size. Thus, he selected re-irradiation with SBRT after receiving information regarding the possibility of serious toxicity due to repeated SBRT and providing informed consent.

The second SBRT was performed 11 months after the first SBRT. A Sengstaken–Blakemore tube was inserted below his diaphragm by laparoscopic surgery before the second SBRT in order to keep his stomach away from the tumor. The balloon was expanded with 200 ml of normal saline. A total dose of 50 Gy in 4 fractions was delivered to the isocenter. The Dmax of the stomach was 48.0 Gy, but we considered that the high-dose region differed from the previous one treated with the first SBRT, based on the change of the stomach shape by the Sengstaken–Blakemore tube. The dose distribution and dose volume histogram of the stomach are shown in Figs. 1b and 2b, respectively. One of four sessions was performed during free-breathing because of his depressed level of consciousness. He was irradiated with verification of tumor reproducibility using a respiratory monitoring system.

**Fig. 4:**
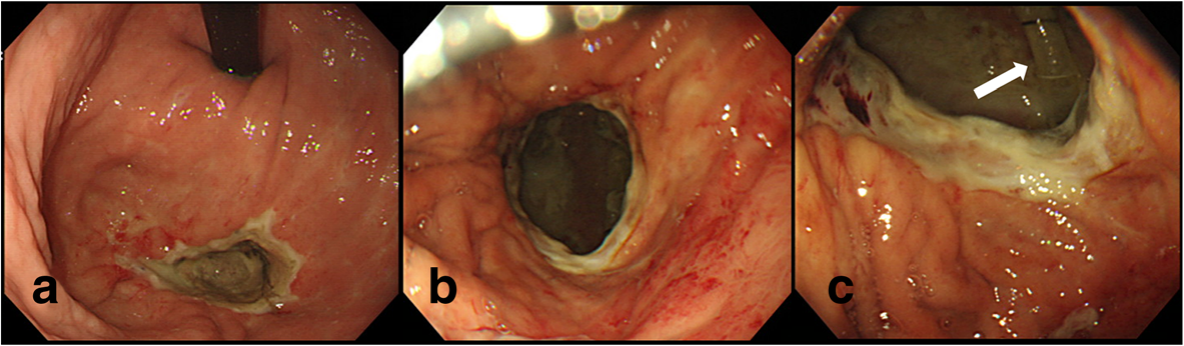
Endoscopic images of stomach after the second stereotactic body radiotherapy. **a** A deep ulcer was observed on the fornix at 1 month after the second stereotactic body radiotherapy. **b**, **c** A gastric perforation and gastropleural fistula were noted at 3 months after the second stereotactic body radiotherapy. The *arrow* shows a drainage tube

One month after the second SBRT, he was hospitalized with melena and anemia. An endoscopy revealed a deep ulcer in the same region as after the first SBRT, and he was treated with a proton pump inhibitor (Fig. 4a). Subsequently, he developed left thoracic empyema, and a tube was inserted into his left thorax for drainage. Two months after the second SBRT, a gastric perforation and gastropleural fistula penetrating his diaphragm were suspected because of food debris being observed in his drainage tube. He was treated conservatively because of inoperability. Three months after the second SBRT, an endoscopy showed a large gastric perforation and gastropleural fistula (Fig. 4b, c), and he died of multiple organ failure with thoracic empyema.

## Discussion

In recent reports, re-irradiation with SBRT for lung tumors previously treated with thoracic radiation therapy resulted in several serious toxicities [4, 6, 7, 10, 11, 17]. Most cases of serious non-lung toxicities were observed in patients with central tumors. Peulen *et al*. reported the following serious toxicities after re-irradiation with SBRT for central tumors: three cases of grade 5 hemoptysis and one case of grade 4 vena cava superior stenosis and grade 4 fistula between the trachea and gastric tube [4]. Kilburn *et al*. reported a case of grade 5 aorta-esophageal fistula after re-irradiation with SBRT for a central tumor previously treated with chemoradiotherapy [10], while Trovo *et al*. reported a case of fatal hemoptysis after re-irradiation with SBRT for central disease [11].

In our case, the patient had a peripheral tumor that was treated twice with SBRT, and consequently developed serious gastric perforation. Referring to the endoscopic images, we suspected that this resulted from the high dose of the second SBRT delivered to the same gastric region as the first SBRT. Although we tried to extend the distance between the stomach and the tumor and to change the high-dose region in the stomach by inserting a spacer to reduce the stomach dose, we could not sufficiently extend the distance because of coalescent resulting from the first SBRT.

Bae *et al*. analyzed the predictors for severe gastroduodenal toxicity in patients treated with SBRT using 33 to 60 Gy (median, 45 Gy) in 3 fractions for abdominopelvic malignancies [18]. Forty patients, including two and one patients with grade 4 gastric perforation and grade 3 gastric ulcer, respectively, were reviewed. The authors suggested that Dmax values of 35 and 38 Gy were respectively associated with 5% and 10% probabilities of severe gastroduodenal toxicity. In our case, the Dmax values in the first and second SBRT were 45.8 and 48.0 Gy, respectively. Considering that the maximum doses per fraction in our case and the predictive doses suggested by Bae *et al*. [18] were similar, the Dmax values in our first and second SBRT were higher than the above doses. We calculated the accumulated dose of the stomach in the first and second SBRT using MIM Maestro^TM^ (version 6.5, MIM Software Inc., Cleveland, OH, USA). Rigid registration was used to create fusion CT images focusing on the fornix of the stomach. The Dmax of the stomach in the summed plan was 83.5 Gy, which was considerably higher than the predictive doses.

Finally, although a detailed case presentation on serious gastric perforation after SBRT for lung tumors has not yet been reported, based on the present case, we conclude that we should carefully evaluate the stomach doses in both the first and second SBRT. Moreover, we should also be cautious of the toxicity after the first SBRT, and should observe the stomach with endoscopy immediately before the second SBRT.

## Conclusions

To the best of our knowledge, this is the first report about a gastric perforation after SBRT for lung tumors. Our case of re-irradiation with SBRT for a lung tumor suggests that it is necessary to pay attention to not only the tumor location in the lung but also to the doses to the normal tissue and any toxicity after initial irradiation.

## Abbreviations

CT: Computed tomography
CYFRA 21-1: Cytokeratin-19 fragment
Dmax: Maximum dose
FDG-PET: Fluorodeoxyglucose-positron emission tomography
SBRT: Stereotactic body radiotherapy
SUVmax: Maximum standardized uptake value
